# Knowledge of insulin use and its determinants among Nigerian insulin requiring diabetes patients

**DOI:** 10.1186/2251-6581-13-10

**Published:** 2014-01-07

**Authors:** Unyime Sunday Jasper, Macmillian Chinonso Opara, Edna Bawa Pyiki, Olayinka Akinrolie

**Affiliations:** 1 Department of Physiotherapy, Jos University Teaching Hospital, Jos, Nigeria; 2 Department of physiotherapy, National Hospital, Abuja, Nigeria

**Keywords:** Insulin use knowledge, Determinants of insulin use knowledge, Insulin-requiring diabetes patients, Nigerian

## Abstract

**Background:**

Intensive insulin therapy is essential in the maintenance of strict glycemic control among insulin requiring patients with diabetes. However this presents a challenge in the face of the complexities associated with insulin use and also taking into consideration the potential dangers associated with inappropriate use. Insufficient knowledge of insulin use can result in preventable complications, adverse patient outcome, poor adherence to therapy and invariably poor glycemic control.

**Methods:**

Insulin requiring diabetes patients (n = 54) attending the 2012 world diabetes day celebration in a Nigerian community were surveyed using a two part questionnaire. Section A elicited information on their demographics characteristics and participation in update courses, and exercise, while section B assessed knowledge of insulin use using the Michigan Diabetes Research and Training Centre's Brief Diabetes Knowledge Test. All participants who had a good grasp of English language or who could understand the contents of the questionnaire when it was explained to them, and were willing to participate in the study were assessed. Descriptive statistics of percentages was computed for the sociodemographic variables, previous education, satisfaction with education, involvement in regular exercise, knowledge of benefit of exercise and correct response to each question in section B. Analysis of variance (ANOVA) and independent t-test was used to determine the influence of sociodemographic variables on insulin use knowledge.

**Results:**

Knowledge of insulin use is poor among insulin requiring patients with diabetes, with majority not conversant with such terms as ketoacidosis, insulin reaction and low blood sugar. Furthermore, they did not know how to modify their insulin dosage in relation to diet, exercise and infections (e.g. flu). Better knowledge of insulin use was associated with age, employment status, level of education attained, how frequent one reads/attends update courses and satisfaction with education received.

**Conclusion:**

Poor knowledge of the causes and prevention of the ketoacidosis, insulin reaction and hypoglycemia increases their risk of developing them, which will invariably lead to poor adherence to insulin therapy. Therefore this study suggests a methodical, continuous and up-to-date tutelage if proper self management in terms of good glycemic control is to be achieved.

## Background

The prevailing burden of communicable and infectious diseases is already heaping enormous pressure on the health care system of developing countries. These, coupled with the increase in the prevalence of non communicable diseases such as diabetes projected to become one of the world's main disabler and killer within the next twenty-five years [1] only intensifies the gravity of the situation. This is because it is estimated that by the year 2030, over 70% of people with diabetes will reside in developing countries [2]. Diabetes mellitus (DM) is a chronic illness and diabetes care is multi-factorial, going beyond just proper glycemic control. While conscientious diabetes care entails continuing care by medical, diet and physical activity experts, patient self-management education is also imperative. This would help prevent acute complications notably among those on insulin therapy and in the long run forestall the threat of long-term complications.

The diabetes control and complications trial (DCCT) and the Epidemiology of Diabetes Interventions and Complications reported that maintenance of strict glycemic control delays the onset and slows down the progression of complications in type 1 diabetes [3,4]. The DCCT clearly showed that intensive insulin therapy (three or more injections per day of insulin or continuous subcutaneous insulin infusion (CSII, or insulin pump therapy) is a key part of improved glycemia and better outcomes [5,6]. Insulin is one of the most commonly prescribed medications in the hospital; it can also be one of the most harmful medications if used inappropriately [7]. For this reason, it is identified as one of five “high alert” medications that have the greatest risk of causing injury to patients because of medication errors by The Joint Commission on Accreditation of Healthcare Organizations [8]. While insulin therapy is key part in management of type 1 diabetes, other types of diabetes mellitus require insulin therapy in its management depending on the balance between insulin secretion and insulin resistance.

Effective usage of insulin in the management of glycemia remains a challenge in developing countries like Nigeria [9]. Several factors contribute to this, chief among which is insufficient knowledge of insulin and diabetes management on the part of health care providers and patients leading to errors in insulin therapy. Consequently, preventable and life-threatening complications such as hyperglycemia and hypoglycemia may ensue. Hypoglycemia, especially in insulin treated patients, is the leading limiting factor in the glycemic management of type 1 and type 2 diabetes [10]. Additional triggering events leading to iatrogenic hypoglycemia include inappropriate timing of short- or rapid-acting insulin such as aspart and lispro in relation to meals [11].

The sole greatest panacea to accurate self-managed insulin therapy and deterrent against complications arising from insulin use is adequate and comprehensive knowledge of the complexities associated with insulin therapy. This is imperative because over the years, the number of insulin types has doubled [7] increasing the chances of medication errors. In a study on self-reported understanding of DM and its treatment among the elderly subjects, it was shown that despite high self-reported understanding of DM and its treatment, 24.5% of subjects made at least one error with regard to their medication treatment [12]. Therefore, in addition to basic knowledge of diabetes which should be transmitted at diagnosis, insulin requiring patients with diabetes demand regular and systematic education throughout their lives. This is to ensure the patient maintains basic knowledge of insulin use and up-to-date awareness of the new principles, techniques of administration, and procedures involved in insulin therapy.

Insufficient knowledge of insulin use can result in preventable complications, adverse patient outcome, poor adherence to therapy and invariably poor glycemic control. Knowledge of the typical daily insulin requirements and basal insulin requirements in type 1 patients is required to avoid insulin overdosing [7]. There is a global paucity of data in literature on knowledge of insulin use among diabetics on insulin therapy, with only a few among health workers [7,13,14]. To date we are unaware of any study that has evaluated knowledge of insulin among diabetics in Nigeria. Considering the risk involved in inappropriate insulin use, attention to evaluating knowledge of insulin use is important because nowadays insulin requiring diabetes patients are encouraged to own insulin delivery kits so as to ensure timely administration of basal insulin. Therefore the aim of this study is to assess knowledge of insulin use among insulin requiring patients with diabetes, with the goal of identifying gray areas for future educational interventions.

## Material and methods

### Sample

This study was a descriptive cross-sectional pilot study which utilized a sampling of convenience to recruit all eligible diabetic patients who attended the 2012 world diabetes day celebration at a diabetes screening centre in Jos, Plateau State, Nigeria.

### Instrument

The instrument utilized in this study was a two-part questionnaire. Section A dealt with patient demographics (age, gender, religion, level of education, occupation/employment status, level of household wealth and family history of diabetes etc.) and the disease (time since diagnosis, type of treatment, regularity on medication and type of diabetes), It also contains questions on their involvement in exercise, whether exercise is beneficial for diabetes, reading/attending update courses and satisfaction with information gathered. Section B assessed knowledge of insulin use using questions relating to insulin use from the Michigan Diabetes Research and Training Centre’s Brief Diabetes Knowledge Test [15], which was created for adults with either type 1 or type 2 diabetes. Fourteen multiple-choice questions assess basic patient knowledge of diabetes, while nine assess patient’s knowledge of insulin use. A reliability coefficient of 0.70 was reported for the general test and insulin use subscale [15].

### Methodology

Approval to carry out this study was sought and obtained from the management of the diabetes screening centre in Jos, Plateau State, Nigeria. Prior to the commencement of the interview, the purpose of the study was thoroughly explained to the participants. All participants who had a good grasp of English language or who could understand the contents of the questionnaire when it was explained to them, and were willing to participate in the study were assessed. Furthermore the research assistants were on hand to attend to any questions arising from the respondents, while they assisted those who could neither read nor write to complete their questionnaires.

It was ensured that the feedback came from them so as to ensure that they understood the questions very well. For those with visual impairments, the questions were read out to them and they provided answers. However, participants who have not previously been diagnosed of diabetes were excluded from the study as were those who could not understand the contents of the questionnaire after it has been explained to them (illiterate or visually impaired). Participants who were under 18 years, mentally or speech impaired were also excluded.

Participants who are insulin alone or along with other medications were required to answer questions nine questions (15–23) on section B of the questionnaire which is for diabetics on insulin therapy [15]. A score of ≥ five was considered satisfactory in this study. Each correct answer was awarded one point and the total score was rated as good (≥5), or poor knowledge (<5), with the maximum score obtainable being 9. Higher scores indicate higher knowledge of insulin use.

### Data analysis

Using SPSS version 17, descriptive statistics of percentages was computed for the sociodemographic variables, previous education, satisfaction with education, involvement in regular exercise, knowledge of benefit of exercise and correct response to each question in section B. Analysis of variance (ANOVA) and independent t-test was used to determine the influence of sociodemographic variables on insulin use knowledge. Proportional differences were explored using chi statistics. Differences were considered significant at an alpha level of 0.05.

## Results

Out of a total of sixty-two (62) questionnaires distributed, 58 were returned and 4 were considered invalid, translating to a response rate of 93.5% (n = 54). A simple majority of the participants in this study were between the age group of 51–60 years (33.3%, n = 18), while those between 21–30 years respectively represented the least age group (5.6% n = 3). Further sociodemographic information is shown in Table 1.

**Table 1:** Sociodemographic variables of participants

**Demographic variables**	**n**	**%**	***X***^***2***^	**p-value**
**Age**				
18-20 years	4	7.4		
21-30 years	3	5.6		
31-40 years	9	16.7		
41-50 years	13	24.1	21.6	0.001
51-60 years	18	33.3		
61-70 years	7	13.0		
**Gender**				
Male	21	38 · 9		
Female	34	61.1	3.630	0.057
**Religion**				
Christianity	46	85.2		
Islam	7	13.0	62.1	0.000
Traditional	1	1.9		
**Marital status**				
Single	6	11.1		
Married	44	81.5	56.3	0.000
Widow/Widower	4	7.4		
**Level of education**				
Primary	16	29.6		
Secondary	5	9.3		
Tertiary	10	18.5	12.2	0.007
None	23	42.5		
**Employment status**				
Unemployed	12	22.2		
Self employed	11	20.4		
Government employed	16	29.6	8.5	0.072
Retired	12	22.2		
Student	3	5.6		
**Level of household wealth**				
Poorest	2	3.7		
Poor	8	14.8		
Intermediate	39	72.2	66.6	0.000
Wealthy	5	9.3		
**Do you have a family history of diabetes?**				
Yes	26	48.1		
No	16	29.6	6.8	0.034
Not sure	12	22.2		
**When were you diagnosed of diabetes?**				
Less than 5 years	27	50.0		
5-10 years	16	29.6		
10-15 years	5	9.3	54.9	0.000
15-20 years	2	3.7		
25-30 years	2	3.7		
>30 years	2	3.7		
**Type of diabetes**				
Type 1	17	31.5	9.0	0.003
Type 2	37	68.5		
**Where were you diagnosed of diabetes?**				
Private hospital/laboratory	15	27.8		
Public hospital	29	53.7	31.0	0.000
Diabetes centre	10	18.5		
**Start insulin therapy**				
Immediately after diagnosis	39	72.2		
1-2 years after	9	16.7	66.1	0.000
3-5 years after	2	3.7		
>5 years after	4	7.4		
**Regular on medication**				
Yes	48	88.9	32.7	0.000
No	6	11.1		
**Exercise regularly**				
Yes	37	68.5	7.4	0.006
No	17	31.5		
**Exercise is beneficial for diabetes**				
Yes	48	88.9		
No	2	3.7	75.1	0.000
Not sure	4	7.4		
**Read articles/attend update courses**				
Not at all	14	25.9		
Rarely	19	35.2		
Often	13	24.1	4.5	0.211
Regularly	8	14.8		
**Satisfied with instruction**				
Very satisfied	7	13.0		
Satisfied	18	33.3	13.4	0.001
Not satisfied	29	53.7		

### Knowledge of insulin use

Overall, Nigerian insulin requiring diabetics knowledge of insulin use was poor, with 61.1% (n = 33) scoring below average with mean knowledge score of 3.3 (SD = 1.9) out of a possible score of 9. An overwhelming majority did not know the sign of ketoacidosis to be vomiting (81.5%, n = 44); that it takes 6–12 hours to have an insulin reaction after taking intermediate-acting insulin (NPH or Lente) (88.9%, n = 47) and the most likely cause of an insulin reaction is heavy exercise (77.8%, n = 41). Table 2 shows the frequency of incorrect answers on the entire questions.

**Table 2:** Percentage incorrect answers on the knowledge of insulin use test

**Item**	**% (n) incorrect**	**Question (correct answer is in bold)**
15	81.5 (44)	Signs of ketoacidosis include:
		a. Shakiness
		b. Sweating
		**c. Vomiting**
		d. Low blood glucose
16	55.6 (30)	If you are sick with flu, which of the following changes should you make
		a. Take less insulin
		b. Drink less liquids
		c. Eat more proteins
		**d. Test for glucose and ketones more often**
17	88.9 (48)	If you have taken intermediate-acting insulin (NPH or Lente), you are most likely to have an insulin reaction in:
		a. 1–3 hours
		**b. 6–12 hours**
		c. 12–15 hours
		d. more than 15 hours
18	61.1 (33)	You realize just before lunch time that you forgot to take insulin before breakfast. What should you do know?
		a. Skip lunch to lower your blood pressure
		b. Take the insulin that you usually take at breakfast
		c. take twice as much insulin as you usually take
		**d. Check your blood glucose level to decide on how much insulin to take**
19	75.9 (41)	If you are beginning to have an insulin reaction, you should
		a. Exercise
		b. Lie down and rest
		**c. Drink some juice**
		d. Take regular insulin
20	46.3 (25)	Low blood glucose may be caused:
		**a. Too much insulin**
		b. Too little insulin
		c. Too much food
		d. Too little exercise
21	59.3 (32)	If you take your morning insulin but skip breakfast, Your blood glucose will usually:
		a. Increase
		**b. Decrease**
		c. Remain the same
13	33.3 (18)	High blood glucose may be caused by:
		**a. Not enough insulin**
		b. Skipping meals
		c. Delaying your snack
		d. Large ketones in your urine
23	77.8 (42)	Which one of the following will most likely cause an insulin reaction
		**a. Heavy exercise**
		b. Infection
		c. Overeating
		d. Not enough insulin

### Sociodemographic determinants of diabetes knowledge

Findings in this study reveal that knowledge significantly increases exponentially as level of education attained (p = 0.006, F = 4.7), with those who had primary school having the lowest scores, while those with secondary and tertiary education scored highest. How often a participant read articles/attended update seminars had a significant effect on knowledge (p = 0.000, F = 64.3), with participants who updated their knowledge regularly or often scoring higher than those who did so often, rarely and not at all. Furthermore those who were very satisfied or satisfied with education received had better knowledge than those who were not satisfied with education received (p = 0.000, F = 66.0). The younger age groups of 18–20 years to 21–30 years respectively were more knowledgeable of insulin use than their older counterparts in the 51–60 years and 61–70 years (p = 0.026, F = 2.8). Furthermore, students scored significantly higher than the self employed, unemployed, retired (p < 0.05, F = 1.74). Table 3 depicts the sociodemographic determinants of insulin use knowledge.

**Table 3:** Sociodemographic determinants of diabetes knowledge

**Variables**	**Mean (SD)**	**F-value**	**p-value**
**Level of education attained**			
Primary	2.0 (0.9)^b^		
Secondary	4.1 (1.8)^a^	4.7	0.006
Tertiary	3.8 (2.0)^a^		
None	2.0 (0.8)^b^		
**Read articles/attend update seminar**			
Regularly	5.4 (0.5)^a^		
Often	5.1 (1.3)^a^	28.8	0.000
Rarely	2.2 (1.2)^b^		
Not at all	2.1 (1.1)^b^		
**Satisfaction with education received**			
Very satisfied	5.0 (1.9)^a^		
Satisfied	4.8 (1.2)^a^	32.9	0.000
Not satisfied	2.0 (1.1)^b^		
**Employment status**			
Government employed	2.8 (1.7)^b^		
Self employed	3.0 (2.2)^b^	2.9	0.031
Unemployed	3.6 (2.0)^b^		
Retired	2.7 (1.7)^b^		
Student	5.3 (0.5)^a^		
**Age**			
18-20 years	5.3 (0.5)^a^		
21-30 years	5.3 (0.6)^a^		
31-40 years	3.3 (2.1)^a^		
41-50 years	3.8 (1.5)^a^	2.8	0.026
51-60 years	2.6 (1.8)^b^		
61-70 years	2.7 (2.1)^b^		

For a particular variable, scheffe post hoc test revealed that means with different superscript are significant at p < 0.05 (superscripts a and b). Means that share the same superscript are not significantly different from each other (p > 0.05).

Knowledge of insulin use was not associated with gender, religious afffiliation, having a family history of diabetes, duration since diagnosis, type of diabetes and where they were diagnosed of diabetes (*p > 0.05*).

## Discussion

Optimal control of DM by insulin dependent diabetes patients requires effective and meticulous self-management in terms of diet, exercise, medication adherence, but the complexities associated with these present challenges to self-managing the disease well. While medical and health professionals will not always be around to guide and influence patients decisions regarding when and how to administer insulin at home, life-long education on administration, complications and benefits of adherence to therapy provides knowledge, skills and confidence for the patient.

There is alarming and widespread poor knowledge of insulin use among Nigerian insulin requiring diabetes patients. For example majority did not know signs of ketoacidosis, cause of an insulin reaction, time it takes to have an insulin reaction after taking intermediate acting insulin and what to do if they develop an insulin reaction. The participants in this study may not be conversant with these terms (ketoacidosis and insulin reaction) possibly because in an attempt to simplify these medical terms to the understanding of the illiterate and semi-illiterate members, educators sometimes miss out on these main terms altogether. This finding is in line with an earlier study which reported poor knowledge of signs of ketoacidosis and cause of an insulin reaction among majority of veterans in the US [16].

Hypoglycemia is the leading limiting factor in the glycemic management of type 1 and insulin-treated type 2 diabetes [10] and may be an obstacle to the use of insulin [17]. In Nigeria, hypoglycemia was the most frequently documented problems encountered by persons on insulin [9]. However, a slight majority of the participants in this study did not know the cause of low blood sugar. Even though teaching people with diabetes to balance insulin use with carbohydrate intake and exercise is necessary may not always be an adequate approach due to natural defects such as hypoglycemia-associated autonomic failure [11], educating them on ways of identifying the signs and preventing hypoglycemia is an essential component of diabetes management for insulin requiring patients with diabetes. It is however heartwarming that a simple majority (66.7%, n = 36) knew that too little insulin could lead to hyperglycemia. Furthermore, that an overwhelming majority (90.7%, n = 49) of them knew the benefits of exercise, but many were not exercising regularly (33.3%, n = 18) would probably mean that knowledge may not necessarily lead to good practice or performance.

Furthermore, majority did not know how to modify their insulin dosage in relation to diet and infectious diseases such as flu. This is consistent with an earlier study which revealed that questions eliciting the highest incorrect answers concerned modifying insulin dosing according to physical therapy [13]. This prevalent poor knowledge is probably because, health care professionals who are supposed to put clients through on the technicalities involved in modification and regulation of insulin dose according to diet, exercise and infectious diseases are also deficient themselves. Previously, a study in US has reported poor insulin-related knowledge among health care professionals [7].

The younger participants were also more knowledgeable than their older counterparts. With the positive influence of employment on knowledge in this study, this finding is probably explained by the fact that students were likewise more knowledgeable than participants who were either government employed, unemployed, self employed and retired. It has been found that older persons with diabetes tend to have less education, worse cognitive function, and more barriers to practicing appropriate self care than their younger counterparts [18,19]. Moreover, because young people are more voracious and inquisitive in search for further enlightenment about their condition, they may consult other reliable sources of information other than that from a health educator. Satisfaction with education received whether update courses or seminars was associated with good knowledge of insulin use. Satisfaction will mean incorporation of information received into their daily routine of proper insulin use, proper dieting, optimal physical activity and avoidance of preventable complications such as hypoglycemia. This will go a long way to improving their attitude towards insulin therapy and diabetes in general, coping strategies and invariably quality of life, and in the long run change their practices to embrace healthier lifestyles along with adhere to insulin therapy.

Also participants who either regularly read or attended seminars on diabetes were more knowledgeable than those who did not. This finding is in line with an earlier study which reported improvement in knowledge with an education course on insulin use [13]. Also, Weeraratne et al. [14] reported improvement in post-test knowledge of insulin therapy among health professionals after a training program. In other to achieve good glycemic control among insulin deficient diabetics, methodical, continuous and up-to-date tutelage is important to aid them on various insulin preparation available, correct injection techniques, appropriate insulin use and in understanding how to alter their insulin in relation to diet and exercise. Furthermore, knowledge of the typical daily insulin requirements and basal insulin requirements in type 1 diabetic patients is required to avoid insulin overdosing [7], under dosing, prevention and prompt recognition of any complications that may arise.

Level of education had a significant effect on diabetes knowledge, with those who have attained secondary or tertiary education scoring higher than those with primary education or no education at all. During didactic sessions in Nigeria, English language is mostly used and even though interpreters are employed occasionally to reach out to the less educated not all of them benefit considering the multiethnic nature of the country. The high illiteracy rate in this study may have contributed to the poor overall knowledge of insulin use. Therefore instructive program designers should seek ways of tackling the hindrance created by illiteracy so as to prevent comprehension bias. Furthermore, since a schooled person may be more probing while being lectured by health professionals than an uneducated one, educators should be more practical and modify their pedagogical skills while dealing with the less educated to ensure their maximal participation and assimilation.

Another important finding in our study is that even though the majority was faithful in their insulin intake, a few were still not regular on medication. One of the reasons attributed to these is the cost of acquiring insulin in Nigeria [9]. While preaching prevention and proper management of diabetes, government and non-governmental agencies alike should also seek ways of subsidizing the cost of insulin and insulin analogues just as it is done for other epidemics; a level which diabetes is approaching. Alternatively, all medications and supplies such as syringes, strips, and meters, related to the daily care of diabetes must also be reimbursed by third-party payers [11].

### Strength and limitations of the study

A limitation of our study is that the sample size was small, even though it should be noted that this was a pilot study. Furthermore, considering that it was a single centre study, the results of our study should be interpreted with caution. However, this study also has its strengths. To our knowledge, this is the first study to utilize a reliable and validated questionnaire to evaluate knowledge of insulin in a population based insulin requiring diabetic sample in an African population. Therefore to build on this, studies with larger sample sizes are advocated.

## Conclusion

Nigerian insulin requiring diabetics possessed poor knowledge of insulin use and are not conversant with some important terms such as ketoacidosis, insulin reaction and hypoglycemia. Poor knowledge of the causes and prevention of the above terms increases their risk of developing them, which will invariably lead to poor adherence to insulin therapy. Furthermore, knowledge of insulin use was influenced by age, level of education, employment status, how frequent one attends seminars/read updates and satisfaction with education received. Professional health care providers are partly to blame for participants being in the dark as to adjusting their insulin dose in relation to diet and infectious diseases. Therefore this study suggests adequate, prompt and frequent education for health professionals who are supposed to enlighten their insulin requiring clients. For the insulin requiring diabetics, a methodical, continuous and up-to-date tutelage is important to achieve proper self management. Finally to reach out to the less and none educated, a more practical approach in addition to modification of pedagogical skills is required of the educators.

## Competing interest

The authors declare that they have no competing interests.

## Authors’ contribution

USJ, MCO and EBP conceived the study. USJ, OA and MCO carried out literature search, data analysis and wrote the first draft. USJ, OA and MCO were involved in the study design. MCO, EBP, OA were involved in data collection, abstraction and interpretation. All authors critically revised the manuscript for intellectual content. All authors have seen and approved the final draft.
